# Psychopathology predicts mental but not physical bariatric surgery outcome at 3-year follow-up: a network analysis study

**DOI:** 10.1007/s40519-022-01463-x

**Published:** 2022-08-27

**Authors:** Alessio Maria Monteleone, Inbal Globus, Giammarco Cascino, Anat Brunstein Klomek, Yael Latzer

**Affiliations:** 1 Department of Psychiatry, University of Campania “Luigi Vanvitelli”, Naples, Italy; 2 School of Public Health, University of Haifa, Haifa, Israel; 3 Maccabi Healthcare Services, Tel-Aviv, Israel; 4 Department of Medicine, Surgery and Dentistry ‘Scuola Medica Salernitana’, Section of Neurosciences, University of Salerno, Salerno, Italy; 5 Baruch Ivcher School of Psychology, Reichman University, Herzliya, Israel; 6 Eating Disorders Institution, Psychiatric Division, Rambam Medical Center, Haifa, Israel; 7 Faculty of Social Welfare and Health Sciences, University of Haifa, Haifa, Israel

**Keywords:** Bariatric surgery, Network analysis, Outcome, Psychopathology, Interpersonal

## Abstract

**Purpose:**

This study aimed to explore the psychopathological variables that may predict bariatric surgery outcomes after 3 years.

**Methods:**

One hundred ninety-six candidates for bariatric surgery completed self-report questionnaires to assess eating attitudes, eating disorder (ED)-related psychopathology, affective symptoms, interpersonal and psycho-social functioning. One-hundred patients repeated this assessment 3 years after bariatric surgery. A network analysis was run including the pre-surgical measurements in the network. A composite score derived from the combination of the most central network nodes, as well as clinical and socio-demographical variables, was included in a multivariate regression analysis with weight loss, ED psychopathology and psycho-social functioning as outcomes.

**Results:**

Depression, stress, and shape concerns were the most central network nodes. The composite network score predicted higher ED psychopathology and worse psycho-social functioning at 3-year follow-up, but not weight loss. Higher age, restricting type of bariatric surgery and higher pre-operative BMI were further predictors of reduced weight loss and greater ED psychopathology.

**Conclusions:**

Affective symptoms and shape concern play a central role in the psychopathology of candidates to bariatric surgery and predict post-surgery ED psychopathology and psycho-social functioning. These variables may allow to identify patients with higher pre-operative risk and in need of further psycho-social interventions.

**Level of evidence:**

III, evidence obtained from well-designed cohort study.

**Supplementary Information:**

The online version contains supplementary material available at 10.1007/s40519-022-01463-x.

## Introduction

Bariatric surgery is a reliable procedure to obtain weight loss and improve metabolic comorbidities in people with severe obesity [1]. This procedure has a notable impact on an individual's day-to-day lifestyle given the modifications that implies on eating attitudes, body image perceptions and interpersonal functioning [2]. In this line, long-term follow-up studies showed that, despite significant weight loss, several psychological aspects are worsened in comparison to pre-operative levels [3, 4]. Recent findings revealed that psychiatric admissions after bariatric surgery are even more common than before surgery [5]. People who had bariatric surgery reported the need for long-term aftercare that addresses social and psychological difficulties [6, 7]. Mental well-being is intertwined with physical well-being, and this is essential to achieve positive outcomes in post-bariatric individuals [2]. As a consequence, exploring psychopathology and identifying the psychopathological variables predicting outcome in candidates for bariatric surgery is a research priority and may allow to outline putative risk factors that characterize individuals in need of early psychological support.

The network theory [8] may allow researchers to target this topic. Indeed, it conceptualizes disorders as the product of symptom interaction instead of the consequence of a latent variable [8, 9] and makes possible to explore psychiatric comorbidity and psychopathology in terms of connections between symptoms, which are usually acknowledged as belonging to different psychiatric disorders according to the current diagnostic manual systems (e.g., the DSM-5). This is helpful to explore the psychopathology of candidates for bariatric surgery. According to a recent meta-analysis [10], binge-eating disorder and depression were the most common mental disorders across people seeking for bariatric surgery; however, these conditions did not predict weight outcome. In this line, several studies investigated psychopathological categories as putative outcome predictors, but their results were contradictory. Indeed, while some studies identified anxiety, depression, and other psychiatric disorders as predictors of poor outcome [11–13], other studies did not replicate those findings [14, 15]. The network analysis explores psychopathology from a dimensional symptom perspective and identifies the most central nodes in the network as the variables which are fundamental to maintain psychopathology and to predict treatment outcome [8, 16]. In addition, it allows to simultaneously assess the interaction of symptoms with factors that have been under-investigated in candidates for bariatric surgery, such as interpersonal problems and dysfunctional eating attitudes, which actually do not represent diagnostic categories but are very common in these individuals [17–20]. Interpersonal problems are associated with different psychiatric disorders [21–25] as well as with psychological distress and global functioning [26] and people with overweight report heterogeneous interpersonal difficulties [27]. A more recent meta-analysis highlighted that people with obesity experience greater insecurity, sadness and more stress following interpersonal situations than healthy controls and report fear of rejection and isolation as possible triggers of abnormal eating [17].

### Aims

The present study attempted to employ the network analysis to explore psychopathology and interpersonal variables and to evaluate their prognostic role.

The first aim of this study was assessing the network structure of psychopathology in candidates for bariatric surgery entering in the network a broad range of symptoms, including eating, affective (stress, depressive and anxious) symptoms, and interpersonal difficulties. These latter include a range of interpersonal problems conceptualized along the dimensions of dominance and affiliation (i.e., social inhibition, assertiveness, hostility, intrusiveness, having minimal feelings of affection, having difficulties supporting others, being excessively caring or dependent). In accordance with a recent review of network findings outlining the centrality of cognitive and psychological symptoms rather than behavioral variables [28] and given the high rate of eating and affective disorders in candidates for bariatric surgery [10], we hypothesized that overvaluation of body shape and weight as well as anxious and depressive symptoms were the most central nodes. The second aim was evaluating whether central network nodes predicted clinical outcomes in those who underwent bariatric surgery. Based on previous network findings [29–31], we hypothesized that the most central nodes would predict weight loss, eating disorder (ED) psychopathology and psychosocial functioning at follow-up. The identification of psychological prognostic factors may promote more personalized and multidisciplinary treatments for candidates to bariatric surgery, highlighting the role of psychological care to help these patients adopting healthy life-style behaviors [32].

## Methods

### Procedure

One hundred ninety-six (60 males, 136 females) candidates to bariatric surgery were consecutively recruited while attending the bariatric surgery committee in Maccabi Health Care and Assuta medical center for pre-surgical psychiatric assessment. Inclusion criteria were (1) age: 18–65 years at the start of the study; (2) Jewish individuals (both sexes), Hebrew speaking, from diverse cultural groups; (3) candidates without diagnosis of psychosis taking any anti-psychotic medications, or acutely suicidal. Before the surgery, bariatric candidates underwent a bariatric committee which included a social worker or a psychologist with specialized training in the bariatric field. They were required to report through a routine clinical interview (and sometimes also their medical files were checked) if they had any history of psychosis or suicide ideation.

The study was approved by the Institutional Ethical Review Board at Assuta Medical Center, Israel, and was carried out in accordance with the Declaration of Helsinki for experiments involving humans. All the participants gave their written consent after being fully informed of the nature and procedures of the study.

### Psychological assessment

Participants were asked to complete the following self-report measures before entering specific treatment programs and at 3-year follow-up.

#### Demographic information and patient history

Participants self-reported age, sex, the highest degree, employment and social status, marital status, and medical comorbidities.

#### *Eating Disorder Examination Questionnaire (EDE-Q)* [33, 34]

The EDE-Q is a 36-item self-report instrument of eating disorder psychopathology including four subscales assessing weight concern, eating concern, shape concern and restraint (EDE-Q global Cronbach’s *α* = 0.78).

#### Depression, Anxiety and Stress Scales (DASS-21) [35]

The DASS-21 is a 21-item self-report instrument designed to assess psychological distress over the past week. The DASS-21 includes three subscales assessing symptoms of depression, anxiety, and stress, as well as an overall score pointing to general distress (DASS-total Cronbach’s *α* = 0.86).

#### Social Adjustment scale-Self-Report (SAS-SR)

The SAS-SR [36] evaluates functioning in six role areas, including work, social and leisure activities, relationships with extended family, role as a marital partner, parental role, and role within the family unit. The 54-item are coded on a five-point Likert scale. An overall score is obtained, as well as scores for each of the six role areas. Higher overall scores indicate greater impairment (SAS-SR total Cronbach’s *α* = 0.62).

#### Inventory of Interpersonal Problems-32 (IIP-32)

IIP-32 [37] is a 32-item self-report measure of interpersonal difficulties which are conceptually organized along the dimensions of dominance and affiliation [38]. It includes 8 subscales (hard to be sociable, hard to be assertive, too aggressive, too open, too caring, hard to be supportive, hard to be involved, too dependent and total interpersonal problems) and a total score. Higher scores point to more severe interpersonal difficulties (IIP-32 total Cronbach’s *α* = 0.82).

#### Family Eating and Activity Habits Questionnaire (FEAHQ) [39]

FEAHQ is a 32-item self-report instrument designed to assess the eating and activity habits as well as obesogenic factors in the overall home environment. It includes five subscales (activity level, stimuli in the environment, eating related to hunger, exposure to healthy food and eating style) and a global score FEAHQ total Cronbach’s *α* = 0.54.

All the questionnaires, except EDE-Q and FEAHQ, have been translated in Israelian by translators fluent in English. To verify that translations captured the original meanings, independent back translation into English was done.

Clinical team provided participants’ weight and height for the calculation of the body mass index (BMI) at baseline and follow-up. The mean percentage Excess Weight Loss (%EWL) is the standard weight outcome measurement in bariatric surgery nomenclature. This calculation is derived from the formula: %EWL = (weight loss/excess weight) × 100, with excess weight being the total preoperative weight minus the ideal weight [40].

After bariatric surgery, the patients were encouraged to have any kind of follow up by a nutritionist, a social worker or a psychologist and a physician but we had no control on their decision.

### Statistical analysis

Differences between initial assessment and follow-up in terms of %EWL, EDE-Q psychopathology, DASS-21 and SAS-SR total scores were calculated through a *t* test for paired samples.

A *t* test for independent samples was used to compare clinical characteristics between patients who completed the follow-up assessment and those who did not participate. To account for multiple tests, Bonferroni correction was applied.

### Network analysis

The variables collected at the pre-surgical assessment were included on subscale level in a network analysis. According to the methodology described by Epskamp et al. (2012), a partial correlational network analysis was performed through R [42], version 3.4.4, using *qgraph* package. The centralityPlot function in *qgraph* was employed to measure the strength centrality index. To address the issue of spurious connections and retain only meaningful associations, a ‘least absolute shrinkage and selection operator’ (LASSO) regularization was applied [43]; that procedure shrinks small partial correlations so that only the most robust partial correlations remain visible [44]. The Extended Bayesian Information Criterion (EBIC) [45], a parameter that sets the degree of regularization/penalty applied to sparse correlations, was set to 0.5 in this analysis. Following the Epskamp et al.’s [46] recommendations, the stability of the network was estimated using the *bootnet* package [47]. First, we estimated the Correlation Stability (CS) coefficient, which is the maximum proportion of the population that can be dropped so that the correlation between the re-calculated indices of the obtained networks and those of the original network is at least 0.7. Epskamp & Fried [48] suggested that 0.25 is the minimum cut-off to consider the network reliable. Then we calculated the accuracy of edge-weights by drawing bootstrapped confidence intervals using nonparametric bootstrapping (nboots = 2500). Finally, we tested whether the centralities of nodes significantly differed from one-another through the bootstrapped centrality difference test.

### Multivariate regression analyses

In accordance with previous studies [49, 50], a composite score was created for central network symptoms. To create this index, we made a composite of the top three central symptoms in the network. We chose these symptoms because they were significantly more central than at least 60% of other symptoms in the network, thereby representing the overall “most” central symptoms.

Multivariate regression analyses tested the ability of the composite score to predict continuous outcomes (the %EWL, the EDE-Q total score and the SAS-SR global score). Multivariate regression analyses were conducted using lavaan package [51] in R, Version 3.6.1 [42]. Age, gender, marital status, social status, pre-surgical BMI, surgery type, the occurrence of nutritional and psychological follow-up were included in the same model as predictors.

## Results

### Clinical characteristics of the study sample

Across the 196 patients screened at the initial assessment, 170 (86.7%) of them underwent bariatric intervention and 100 (51%) of them completed the 3 years follow-up after the intervention: 68 (68%) of these underwent the sleeve gastrectomy or the gastric banding (that are the “restrictive” procedures), 32 (32%) underwent the malabsorptive or the mixed techniques (i.e. the Roux-en-Y gastric bypass or one anastomosis gastric bypass). At the time of pre-surgery assessment, mean age of participants was 40.79 ± 5.34; most of them were female (*n* = 136, 69.4%), married (*n* = 144, 73.4%), with a medium–high social status (*n* = 102, 52%). After bariatric surgery, 83% of patients followed a nutritional follow-up and 31% a psychological one.

Comparison of patients who completed the follow-up assessment (*n* = 100) with those who did not (*n* = 76) revealed no significant differences in anthropometric variables (pre-operative BMI), age and clinical variables (EDE-Q, DASS-21, IIP-32, SAS-SR, FEAHQ total and sub-total scores). Thus, the subgroup of patients who completed the follow-up can be considered representative of the overall sample.

The *t *test for paired samples showed that all the assessed variables (i.e. BMI, ED psychopathology, affective symptoms and psycho-social functioning) showed a significant improvement at 3-year follow-up compared to pre-surgical assessment (Table 1). The post-hoc power analysis showed that the present sample size had a power of 0.99 to detect a medium effect size at an alpha value of 0.05.

**Table 1:** Clinical characteristics of participants with obesity (*n* = 100) before (baseline) and 3 years after bariatric surgery (3-year follow-up)

	Baseline (mean ± SD)	3-Year follow-up (mean ± SD)	*t*	*p*
BMI	41.05 ± 5.46	29.81 ± 5.35	27.43	< 0.001^a^
SAS-SR work	1.72 ± 0.56	1.62 ± 0.62	1.05	0.295
SAS-SR social	2.12 ± 0.53	1.92 ± 0.44	3.02	0.003^a^
SAS-SR relationship	2.19 ± 0.74	1.97 ± 0.56	2.85	0.006
SAS-SR family	1.91 ± 0.55	1.79 ± 0.57	1.61	0.111
SAS-SR total	1.81 ± 0.35	1.70 ± 0.34	3.51	< 0.001^a^
EDE-Q restraint	1.99 ± 1.36	1.61 ± 1.48	1.73	0.08
EDE-Q eating	1.65 ± 1.28	1.15 ± 1.18	2.59	0.01
EDE-Q shape	3.89 ± 1.31	2.05 ± 1.52	10.03	< 0.001^a^
EDE-Q weight	3.72 ± 1.32	2.13 ± 1.57	8.53	< 0.001^a^
DASS depression	3.56 ± 3.96	2.03 ± 3.02	3.43	< 0.001^a^
DASS stress	5.11 ± 4.44	2.88 ± 3.09	4.66	< 0.001^a^
DASS anxiety	3.49 ± 3.54	1.62 ± 2.05	4.34	< 0.001^a^

*BMI* body mass index, *SAS-SR* Social Adjustment Scale-Self-Report, *EDE-Q* Eating Disorder Examination-Questionnaire, *DASS* Depression Anxiety and Stress Scale

^a^Significant results following Bonferroni corrections

### Network analysis

**Fig. 1:**
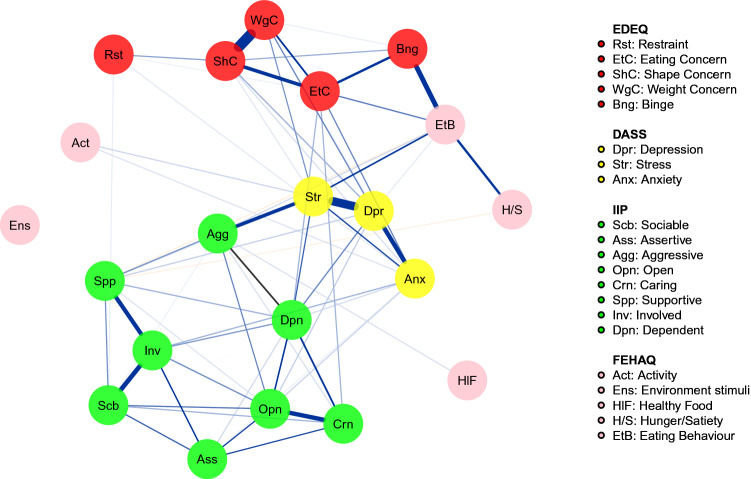
Estimated network of candidates to bariatric surgery

**Fig. 2:**
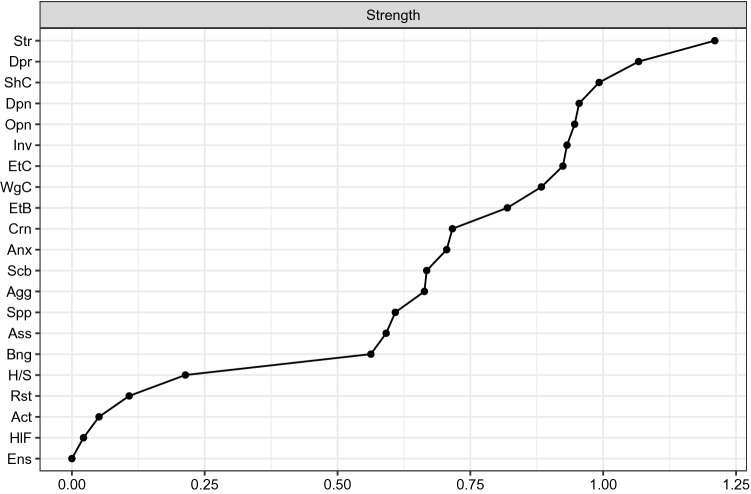
Plot of the strength centrality indices of each network node. *Act* activity, *Agg* aggressive, *Anx* anxiety, *Ass* assertive, *Bng* binge, *Crn* caring, *Dpn* dependent, *Dpr* depression, *Ens* environment stimuli, *EtB*, eating behavior, *EtC* eating concern, *H/S* hunger/satiety, *HlF* healthy food, *Inv* involved, *Opn* open, *Rst* restraint, *Scb* sociable, *Shc* shape concern, *Spp* supportive, *Str* stress, *Wgc* weight concern

The network is reported in Fig. 1 and its strength centrality indices are plotted in Fig. 2. The bootstrapped confidence intervals of estimated edge-weights are reported in Supplementary Fig. 1. The CS for the strength centrality index was 0.44.

The node strength was highest for DASS stress (*M* = 1.21), DASS depression (*M *= 1.07), EDEQ shape concern (*M* = 0.99) and IIP32 interpersonal-dependent functioning (*M* = 0.95). The lowest values were detected for FEAHQ stimuli in the environment (*M* = 0), FEAHQ exposure to healthy food (*M* = 0.02) and FEAHQ activity level (*M* = 0.05). According to the strength centrality difference test (Supplementary Fig. 2), the centrality of the DASS stress, DASS depression and the EDEQ shape concern nodes were significantly higher than the 80 and 70% of the other estimates.

### Regression analysis

**Fig. 3:**
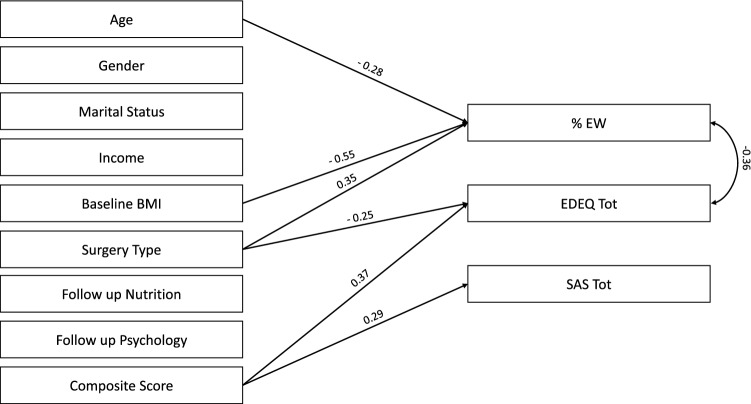
Multivariate regression analysis in bariatric patients who completed the 3-year follow-up. Significant standardized beta values are reported on the arrows

The multivariate regression analysis was run in the 100 patients who completed the 3-year follow-up. The results are represented in Fig. 3. The post hoc power analysis showed that the present sample size had a power of 0.94 to detect a small to medium effect size (*w* = 0.20) at an alpha value of 0.05.

At 3-year follow-up, a greater psycho-social functioning (SAS-SR total score) impairment was positively predicted from the composite score (*ß* = 0.29, *p* = 0.002, higher pre-operative psychopathology was associated with worse psycho-social functioning). The ED psychopathology was positively predicted from type of surgery (*ß* = − 0.25, *p* = 0.02, restrictive interventions were associated with more severe ED psychopathology) and the composite score (*ß* = 0.37, *p* < 0.001, higher pre-operative psychopathology was associated with more severe ED psychopathology). The %EWL was negatively predicted from age (*ß* = − 0.28, *p* = 0.002) and baseline BMI (*ß* = − 0.55, *p* < 0.001, younger age and lower baseline BMI were associated with higher weight loss) and positively predicted from the surgery type (*ß* = 0.35, *p* < 0.001, the “blocking” procedures—the bypass—were associated with higher weight loss). The %EWL and the ED psychopathology were negatively associated to each other (*ß* = − 0.36, *p* = 0.001, lower weight loss was associated with more severe ED psychopathology). No association emerged between SAS-SR total score and both %EWL and ED psychopathology scores.

## Discussion

The present study produced three main findings. First, as expected, people undergoing bariatric surgery showed a significant reduction in body weight and significant improvement in ED-related psychopathology and psycho-social functioning at 3-year follow-up. Second, a network analysis including pre-surgical eating-related and general psychopathological symptoms and interpersonal variables showed that stress, depression, and shape concern were the most central network nodes. Third, the most central network nodes were included in a composite score and higher levels of this score predicted more severe ED psychopathology and worse psycho-social functioning at 3-year follow-up.

Our findings display that people seeking bariatric surgery show a network composed of problematic eating behaviors, ED psychopathology, affective symptoms, and interpersonal difficulties. In the light of the network theory [8], these symptoms may interact and produce feedback loops that maintain the psychopathology structure in these individuals regardless of the occurrence of defined psychiatric conditions. The network *hysteresis* model [8] proposed that a network composed of stable connections may represent the subjects’ vulnerability to external factors (i.e., stressors) which promote the symptoms’ activation and the development of those conditions that we phenomenologically recognize as categorical psychiatric disorders [8, 52]. However, longitudinal studies are needed to confirm this hypothesis. Consistently with literature [10] showing that binge-eating disorder and depression are the most common psychiatric disorders in people seeking for bariatric surgery, the current network showed depressive symptoms, stress, and shape concern as the most central nodes. Therefore, in accordance with our first hypothesis, these findings highlight the importance of stress, depressive symptoms, and body image concerns in people with obesity seeking for bariatric surgery.

One of the most innovative aspects of this study is the inclusion in the network of interpersonal difficulties and dysfunctional eating behaviors. In accordance with literature showing a high occurrence of dysfunctional interpersonal functioning and eating attitudes in candidates for bariatric surgery [2, 17], both groups of nodes were connected to the other nodes related to affective symptoms and ED psychopathology. Although causality cannot be drawn from this network, the observed connections are coherent with literature in people with EDs and with obesity [53–55] showing that interpersonal difficulties have an impact on negative affect and low self-esteem which may lead to ED symptoms. Across interpersonal variables, the most central node was the dependent subscale, which points to excessive worries about people reactions and judgments. This is consistent with the higher levels of social inhibition, negative emotions related to interpersonal situations and rejection sensitivity found in people with obesity compared to normal weight groups [17, 53, 56]. The nodes indicating abnormal eating behaviors include dysfunctional eating habits (i.e., eating while watching tv or emotional eating), frequency of physical activity, eating in response to internal hunger and satiety cues and being exposed to an obesogenic environment. Surprisingly, these nodes showed low centrality in the network: this may reflect the lack of patients’ knowledge relative to the association of these behaviors with affective symptoms, shape and weight concerns and interpersonal problems. Otherwise, it is possible that abnormal eating behaviors may be more directly related to other variables that this study was not designed to assess (e.g., self-esteem, emotion regulation difficulties).

The second aim of this study was to identify the predictive factors of surgery outcome. One hundred patients underwent bariatric surgery and were screened after 3 years showing significant reduction in their weight and significant improvements in the severity of ED psychopathology and psycho-social functioning. Literature studies assessing weight outcome in post-bariatric individuals reported significant reduction in body weight also in longer follow-up assessments [57, 58]. Regarding the psychological outcome, some long-term studies found increased levels of anxiety, depression, neuroticism, fear of intimacy and reduced self-esteem at follow-up [3, 4] while others observed mental health improvement [57]. In accordance with our second study hypothesis, the most central nodes of the network (stress, depressive symptoms and shape concern) were combined in a composite score which positively predicted the ED psychopathology and the psycho-social functioning at 3-year follow-up. This is in line with a recent study [59] which outlined that bariatric patients with suboptimal weight loss reported heightened levels of depression and ED-related psychopathology. On the other hand, this composite score did not predict weight outcome which, in line with literature [60–62], was positively predicted from younger age and lower BMI at the time of surgery and from the type (the “blocking” procedures) of intervention. Remarkably, the employment of a multivariate regression model allowed us to simultaneously assess the effects of many psycho-social and therapeutic variables and to consider the effects of the co-variation between the outcomes. The predictive role of the composite score supports the network theory [16], which describes the most central nodes as potential predictors of longitudinal trajectories of psychopathology. Therefore, it seems likely that affective symptoms and ED-specific psychopathology are central in the maintenance of psychopathology and should be therapeutically addressed to promote better outcomes after bariatric surgery.

### Strength and limits

The main strengths of this study are (a) the inclusion of interpersonal problems as variables in the network; (b) the assessment of a wide range of predictors and different outcomes (weight, psychopathology, and psycho-social functioning) and their co-variance; (c) the long-term follow-up (3 years), that is necessary to overcome the initial phase of mental and physical improvement usually occurring in most of these patients [63, 64].

Limitations of the study need to be acknowledged also. First, the relatively high drop-out rate that reduces the reliability of follow-up results. However, people who completed the follow-up assessment did not differ in many psychopathological variables from those who dropped out after surgery. Second, no personality measures were included in the network despite previous findings suggesting their relevance [65, 66]. Third, a higher CS of the strength centrality index would be preferable to support the stability of the network: the small size of the network sample may have affected this parameter. Fourth, a control group was not enrolled and a population specifically seeking surgical treatment for obesity participated into the study, thus reducing the generalizability of the findings to other obese groups such as those not seeking treatment or seeking non-surgical intervention.

## Conclusions

The network analysis provides a different conceptualization of psychopathology in candidates for bariatric surgery, highlighting the centrality of depressive symptoms and stress as well as of body shape concern and the occurring of connections between interpersonal difficulties and eating and affective symptoms. Our findings confirm the validity of the most central network nodes to predict outcomes, thus demonstrating the utility of network analysis to understand the prognostic value of psychopathology. In addition, a composite score combining pre-surgical levels of depressive symptoms, stress and shape concern may represent a putative prognostic factor to be evaluated during the pre-surgery psychiatric assessment to identify patients at risk of worse outcomes and to provide them pre-operative psychological and nutritional support. Overall, this study supports the need to provide a multidisciplinary approach [67] including psychiatric and psychological interventions to people undergoing bariatric surgery [2]. These approaches should address shape concerns and depressive symptoms. Clinicians may consider the possibility to improve patients’ awareness of the connections between abnormal eating behaviors and affective and interpersonal problems. Furthermore, the centrality of a highly dependent and rejection sensitivity interpersonal style, as well as its connections with affective and eating symptoms, may suggest the possibility to apply well-validated and efficacious interventions addressing these targets in people with EDs (i.e. interpersonal psychotherapy [68] or the New Maudsley Approach [69]) also to individuals with severe obesity seeking for bariatric surgery.

### What is already known on this subject?

The long-term psychological outcome of bariatric surgery is not so adequate as the weight loss. The prognostic role of categorical psychiatric diagnoses is not clear. The network analysis offers a new approach to explore the predictive role of psychopathology.

### What does this study add?

Network analysis allows to explore psychopathology of candidates to bariatric surgery from a dimensional perspective also considering abnormal eating attitudes and interpersonal problems. Shape concern, depression and stress play a central role to maintain psychopathology and their combination results in a pre-operative risk factor which may be helpful to identify patients with worse long-term outcome after surgery and worthy of a multidisciplinary intervention.

## Supplementary Information

Below is the link to the electronic supplementary material.

Supplementary file1 (PDF 38 KB)

Supplementary file2 (PDF 12 KB)

## Data Availability

The data that support the findings of this study are available from the corresponding author upon reasonable request.
