# Supplementary figures and images for: Psychopathology predicts mental but not physical bariatric surgery outcome at 3-year follow-up: a network analysis study
Source: Eat Weight Disord. 2022 Aug 27;27(8):3331–40. doi: 10.1007/s40519-022-01463-x (PMC9803758; doi:10.1007/s40519-022-01463-x)

● Bootstrap mean    ● Sample

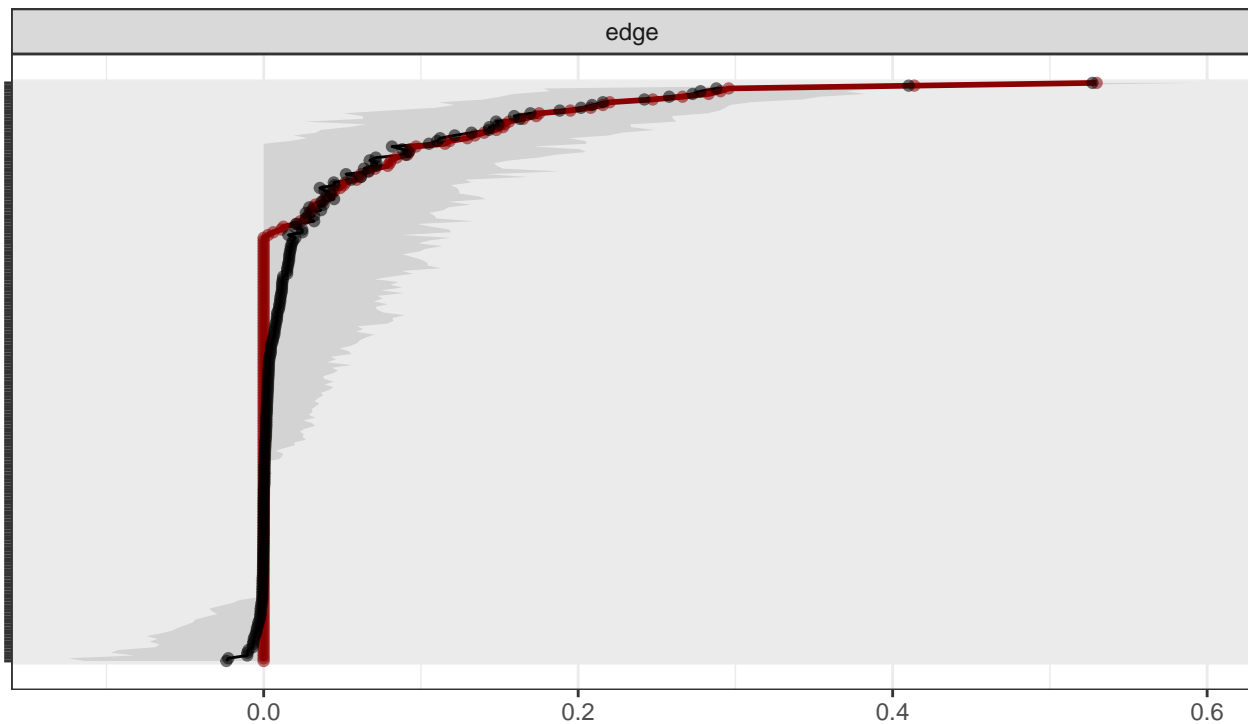

Supplement: Supplementary file 1 — Supplementary file1 (PDF 38 KB) [file 40519_2022_1463_MOESM1_ESM.pdf]

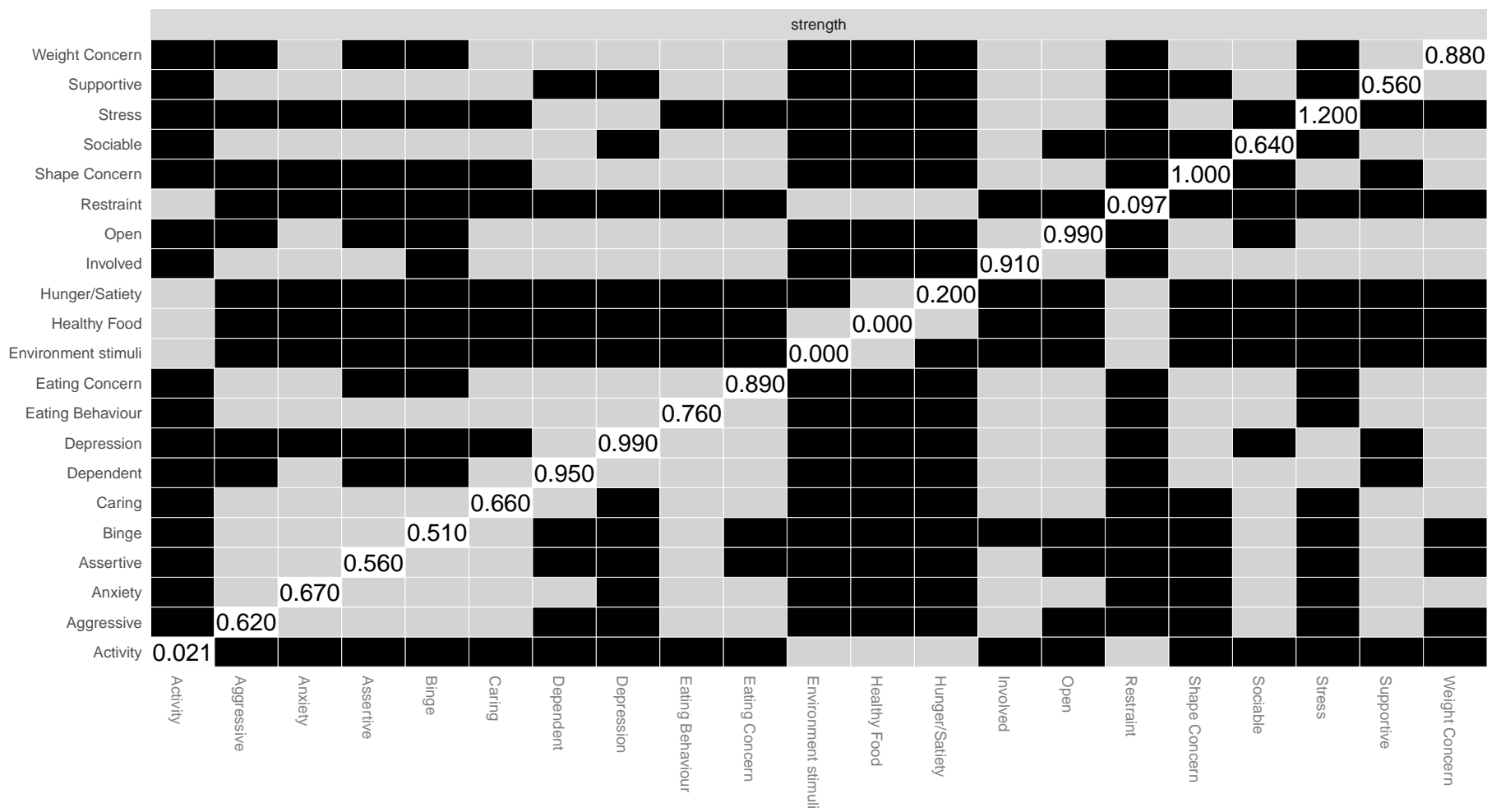

Supplement: Supplementary file 2 — Supplementary file2 (PDF 12 KB) [file 40519_2022_1463_MOESM2_ESM.pdf]
